# A panoramic perspective: application prospects and outlook of multimodal artificial intelligence in the management of diabetic retinopathy

**DOI:** 10.3389/fpubh.2025.1724001

**Published:** 2026-01-16

**Authors:** Chun Liu, Yu Duan, Hao Wu, Junguo Duan

**Affiliations:** 1 Eye College of Chengdu University of TCM, Chengdu, Sichuan, China; 2 Ineye Hospital of Chengdu University of TCM, Chengdu, Sichuan, China; 3 Eye Health with Traditional Chinese Medicine Key Laboratory of Sichuan Province, Chengdu, Sichuan, China

**Keywords:** artificial intelligence, data fusion, diabetic retinopathy, multimoda, precision medicine

## Abstract

Diabetic retinopathy (DR) is a leading cause of blindness among the working-age population, and its management is challenged by the disease's inherent heterogeneity. Current management paradigms, based on standardized grading, are inadequate for addressing the significant inter-patient variability in disease progression and treatment response, thereby limiting the implementation of personalized medicine. While artificial intelligence (AI) has achieved breakthroughs in unimodal analysis of retinal images, the single dimension of information fails to capture the complete, complex pathophysiology of DR. Against this backdrop, multimodal AI, capable of integrating heterogeneous data from multiple sources, has garnered widespread attention and is regarded as a revolutionary tool to overcome current bottlenecks and achieve a panoramic understanding for the management of each patient. This review aims to systematically explore the frontier research and developmental potential of multimodal AI in DR management. It focuses on its data sources, core fusion technologies, and application framework across the entire management workflow. Furthermore, this review analyzes future challenges and directions, with the goal of providing a theoretical reference and guidance for the advancement of precision medicine in DR.

## 1 Introduction

Diabetes mellitus (DM) has become a severe global public health challenge. According to predictions from the International Diabetes Federation, the number of patients with DM worldwide will increase to 783.2 million by 2045 (1). A commentary in The Lancet noted that the effectiveness of DM prevention and control over the next 20 years will profoundly impact population health and life expectancy for decades to come (2). As one of the most common microvascular complications of DM with a significant risk of blindness, diabetic retinopathy (DR) and its management are critical components of the overall DM care system. A recent meta-analysis revealed a global DR prevalence of 22.27%, with the prevalence of vision-threatening DR (VTDR) reaching 6.17% (3).

However, the impact of DR extends far beyond vision impairment alone. As a crucial marker of systemic microvascular disease, its presence is closely associated with an increased risk of severe adverse events, including cardiovascular disease, cognitive dysfunction, and premature mortality (4, 5). These comorbidities collectively pose a serious threat to an individual's quality of life, ability to work, and overall wellbeing. Consequently, implementing early diagnosis and timely treatment is not only key to halting the progression of retinopathy and preventing vision loss but also fundamental to reducing the risk of related systemic complications and improving long-term patient health outcomes (6).

Current DR management models, however, face immense challenges due to the disease's significant individual heterogeneity. Clinically, even among patients at the same stage, disease progression trajectories vary dramatically: some remain stable for long periods, while others deteriorate rapidly. Although the duration of DM and metabolic control are recognized as key risk factors, they fall short of fully explaining the vast differences in disease evolution among individuals or why some patients respond poorly to existing treatments. This inherent heterogeneity renders the “one-size-fits-all” management strategy based on traditional grading standards inadequate. Therefore, a paradigm shift from “standardized grading” to “personalized management” is urgently needed in clinical practice. The core of this shift lies in achieving precise risk stratification, dynamic disease progression prediction, and individualized treatment strategy guidance to address the complexity and uncertainty of DR (7).

Against this background, the rise of artificial intelligence (AI), particularly deep learning, presents a revolutionary opportunity to achieve efficient and precise DR management (8, 9). Among these technologies, multimodal AI has attracted widespread attention for its unique integrative analytical capabilities. It aims to construct a more comprehensive, three-dimensional, and dynamic digital model for each patient by fusing data of different types from various sources (10). This approach has the potential to profoundly characterize an individual's unique pathophysiological state and capture complex associations hidden within unimodal information, thereby providing a solid data foundation for accurate risk prediction and decision support. This represents not only a deepening of existing diagnostic capabilities but also a profound transformation toward achieving full-cycle, all-encompassing personalized management.

In view of this, this review aims to survey the frontier explorations and future potential of multimodal AI in DR management. This paper will first introduce the multimodal data sources used to construct a “panoramic perspective” of DR. Second, it will explore the core AI fusion technologies for achieving efficient data integration. Based on this, it will focus on the application potential and implementation pathways of multimodal AI across the entire DR management workflow—from early warning, precise staging, and dynamic prediction to personalized treatment decisions. Finally, this paper will analyze the challenges currently facing the field and look forward to its future directions, with the aim of providing a reference for research into intelligent DR diagnosis and treatment from a multidisciplinary perspective.

## 2 Multimodal data sources for a panoramic management of DR

The core prerequisite for achieving a “panoramic” management of DR is to move beyond the limitations of single information sources toward a comprehensive analytical framework that integrates multi-dimensional, multi-source data. The necessity of this shift is rooted in an ever-deepening understanding of DR pathophysiology: DR is no longer considered a purely vascular disease but a complex pathological process involving dysfunction of the entire neurovascular unit (11). Furthermore, a series of subclinical structural and functional changes occur in the retina long before the appearance of clinically visible microaneurysms (12). These findings collectively reveal that a truly effective management model must be able to capture and integrate these multi-level pathological changes. Therefore, constructing a comprehensive database that covers retinal macro-morphology, micro-structure, microcirculatory function, systemic risks, and even genetic background is the first step toward personalized management and the foundation upon which multimodal AI can exert its powerful analytical and predictive capabilities. This paper will systematically review the data foundations that constitute this panoramic model, categorized into ophthalmic imaging, systemic indicators, and emerging data sources.

### 2.1 Ophthalmic imaging data

**Figure 1:**
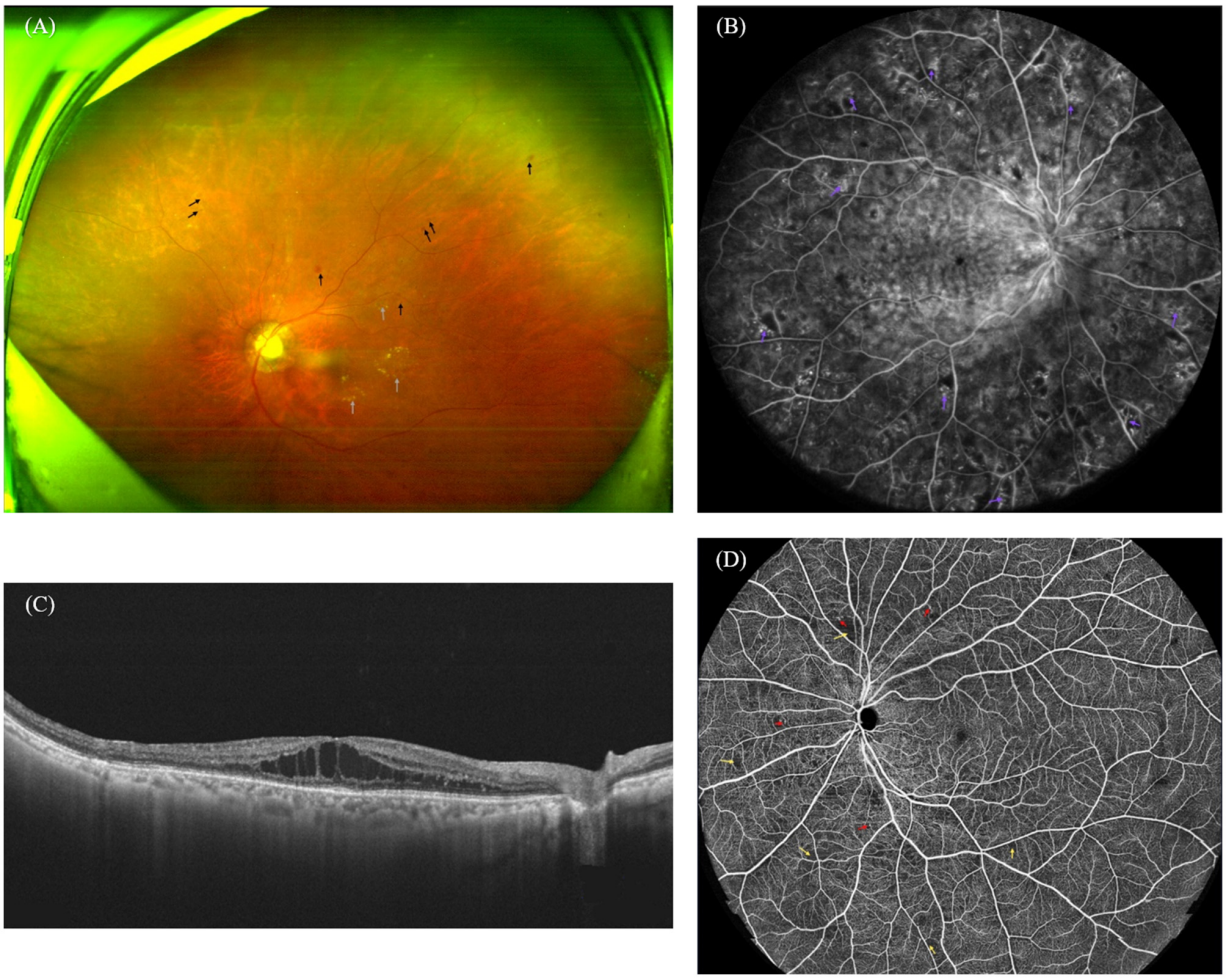
Representative multimodal imaging features of diabetic retinopathy. **(A)** Ultra-widefield color fundus photography (UWF-CFP) revealing scattered retinal hemorrhages (black arrows) and exudates (gray arrows). **(B)** Fluorescein angiography (FFA) demonstrating numerous microaneurysms, patchy areas of capillary non-perfusion, and intraretinal microvascular abnormalities (IRMA, purple arrows). **(C)** Optical coherence tomography (OCT) B-scan exhibiting cystoid macular edema (CME). **(D)** OCT angiography (OCTA) highlighting scattered microaneurysms (red arrows) and areas of capillary non-perfusion (yellow arrows).

Ophthalmic imaging is the cornerstone of DR diagnosis, grading, and follow-up. The advancement of modern imaging technologies allows for non-invasive, high-resolution observation of the retina from multiple dimensions. The application of AI has further unlocked the potential of this imaging data, enabling the extraction of quantitative biomarkers far beyond the range of human visual recognition and facilitating an analytical leap from macro-structure to micro-function and from static assessment to dynamic prediction. A comprehensive visualization of these key pathological features across different imaging modalities is presented in Figure 1.

#### 2.1.1 Color fundus photography

Color fundus photography (CFP) is the most fundamental and widely used imaging technique in DR screening and clinical research (13). By capturing a two-dimensional color image of the posterior pole of the retina, it provides a direct visualization of the classic pathological features of DR, such as microaneurysms, hemorrhages/blots, hard exudates, cotton wool spots, venous beading, intraretinal microvascular abnormalities, and neovascularization. The precise segmentation and feature extraction of these classic lesions form the basis of AI applications in DR (14).

However, traditional seven-field or single-field CFP has a significant limitation: its limited field of view, which primarily covers a 30°-50° area of the posterior pole, neglecting the expansive peripheral retina. A growing body of evidence indicates that predominantly peripheral lesions (PPLs) are an independent and important predictor of the risk of DR progression (15). A key study found that patients with PPLs had a 3.2-fold higher risk of DR progression and a 4.7-fold higher risk of proliferative DR (PDR) than those without peripheral lesions (16). In this context, ultrawide-field (UWF) imaging technology has emerged. UWF can capture an area of up to 200° (approximately 80% of the total retinal surface area) in a single image, clearly visualizing the previously difficult-to-observe periphery and providing a powerful tool for assessing PPLs (17). Studies have shown that UWF imaging has high consistency with traditional seven-field photography within the ETDRS 7-field area, with its core added value lying in its ability to identify and quantify predominantly peripheral lesions outside the traditional fields (18).

Beyond the identification of classic lesions, the quantitative analysis of subclinical retinal geometric features provides important biomarkers for the very early detection of DR (19, 20). Increased retinal venular caliber has been confirmed as a key early indicator. A large-scale meta-analysis confirmed that wider venular caliber is independently associated with the future risk of type 2 DM (21) and is more pronounced in patients with DR (22). Increased vascular tortuosity reflects a state of local ischemia, and recent research further points out that the tortuosity of branch retinal arteries is particularly closely associated with the genesis and severity of DR (23). As a measure of the complexity of the vascular network, a decrease in fractal dimension reflects a simplification of the vascular pattern caused by capillary occlusion. Prospective studies have confirmed that a lower baseline fractal dimension value is an independent predictor of future incident DR (24).

#### 2.1.2 Fundus fluorescein angiography

For decades, fundus fluorescein angiography (FFA) has been the gold standard for evaluating retinal vasculature in DR (25). By intravenously injecting sodium fluorescein, FFA dynamically displays the retinal vascular network with high contrast, enabling the sensitive detection of key pathological features such as microaneurysms, neovascularization, capillary non-perfusion areas, and vascular leakage. This information is crucial for the precise grading, diagnosis, and treatment decisions in DR (26, 27). FFA can clearly distinguish between intraretinal microvascular abnormalities and neovascularization and can locate the source of leakage causing macular edema to guide precise laser therapy (28).

Despite its limitations, such as its invasive nature, the rich dynamic vascular information provided by FFA (e.g., quantifiable metrics like non-perfusion area and leakage index) serves as an extremely valuable data source for AI models. Integrating these functional imaging features into AI models has the potential to surpass lesion identification based on static CFP, thereby enhancing the application potential of AI in precision DR management (29).

#### 2.1.3 Optical coherence tomography

Compared to CFP, which provides a two-dimensional planar view of the retina, optical coherence tomography (OCT) presents its fine three-dimensional cross-sectional anatomy. Using the principle of low-coherence interferometry, OCT performs non-invasive tomographic scans of the retina, clearly displaying its various layers with micron-level resolution. One of its core applications in DR management is the precise quantification of diabetic macular edema (DME). DME is the leading cause of vision loss in patients with DR and is characterized by the breakdown of the blood-retinal barrier and fluid accumulation in the macular region (30). OCT can accurately measure central macular thickness and perform localization and quantitative analysis of intraretinal or subretinal fluid in the macular area (31, 32). This is not only the gold standard for DME diagnosis but also a core objective metric for evaluating the efficacy of treatments such as anti-VEGF therapy (33, 34).

Beyond quantifying DME, OCT also has an irreplaceable value in revealing early neurodegeneration in DR. Retinal neurodegeneration is considered a key event in the early pathogenesis of DR and may even precede the appearance of microvascular lesions (35). As an extension of the central nervous system, the retina provides a unique window for observing neuronal damage that may reflect systemic neurological conditions. OCT can precisely segment and measure the thickness of the retinal nerve fiber layer and the ganglion cell-inner plexiform layer complex. Changes in the thickness of these inner retinal layers are important structural parameters for early DR detection (36, 37). Numerous studies have confirmed that progressive thinning of the retinal nerve fiber layer and the ganglion cell-inner plexiform layer occurs in the early stages of DR, and even in the pre-diabetic state, and that the degree of thinning is closely related to the severity of DR and future visual function impairment (38, 39).

Furthermore, the quantitative analysis of retinal reflectivity using OCT has emerged as a novel method for revealing microstructural and compositional changes in early DR. Studies have confirmed that in DM patients without clinical DR, the reflectivity of the outer retina (especially the ellipsoid zone) is already reduced (40), while changes in the reflectivity of the inner retina (ganglion cell layer) are also closely associated with neurodegeneration (41). These emerging OCT biomarkers provide AI models with richer, quantifiable structural information, opening up new avenues for the very early detection of DR.

#### 2.1.4 OCT angiography

Optical Coherence Tomography Angiography (OCTA) represents a major technological advance in ophthalmic imaging in recent years. Building on OCT, it achieves non-invasive, layer-specific, three-dimensional visualization of retinal and choroidal blood flow by analyzing the decorrelation signal generated by the movement of blood cells between consecutive B-scans (42). Compared to traditional FFA, OCTA does not require the injection of a contrast agent, is rapid, and provides layer-specific blood flow information that is unattainable with FFA (43).

OCTA provides crucial microcirculatory functional information for a panoramic assessment of DR. In the pathophysiology of DR, capillary non-perfusion is considered a core event driving disease progression (11). OCTA can precisely delineate and quantify the size, morphology, and spatial distribution of non-perfusion areas, and its distribution characteristics show significant differences among patients with varying degrees of DR severity (44). Notably, morphological changes in the foveal avascular zone in the macula have been confirmed as powerful predictors of DR progression and visual prognosis (45, 46). Additionally, OCTA can calculate metrics such as vascular density and perfusion density in different regions and at different depths (47). Prospective studies have found that a lower baseline peripapillary vascular density is an independent predictor for the incidence of DR and progression to referable DR over 2 years (48).

### 2.2 Systemic data

As a local microvascular complication of DM in the eye, the pathophysiological process of DR is closely related to the patient's overall systemic condition. The onset and progression of DR are not only influenced by local factors but are also intricately intertwined with long-term metabolic disorders, comorbidities, and treatment adherence. Therefore, any model that detaches from the systemic context and analyzes ophthalmic images in isolation has inherent limitations, as it cannot fully characterize the complete disease state, thereby limiting the accuracy of risk prediction. To construct a “panoramic perspective” that can accurately reflect the full picture of the disease, incorporating systemic data into the analytical framework is a necessary prerequisite for achieving a comprehensive and dynamic assessment.

#### 2.2.1 Electronic health records

The rich, longitudinal clinical information contained in Electronic Health Records (EHRs) serves as a critical bridge connecting ocular pathology with systemic status and constructing a “panoramic perspective.” Among the numerous systemic data types, several categories are particularly crucial for the precise management of DR. First, glycemic control indicators, represented by glycated hemoglobin as the gold standard for assessing long-term blood sugar levels, have been confirmed as the strongest clinical risk factor for the onset and progression of DR (49). Second, metabolic indicators such as blood pressure and lipids are also recognized as independent risk factors; effective control of hypertension and hyperlipidemia can significantly delay the progression of DR (50). Furthermore, the duration and type of DM provide a baseline risk context, with a longer duration typically correlating with a higher prevalence and severity of DR (51), and with type 1 and type 2 DM exhibiting differences in DR presentation and complications (52). Concurrently, systemic comorbidities, especially diabetic kidney disease, which shares a highly homologous microvascular pathogenesis with DR, often herald an accelerated deterioration of DR (53). Finally, the patient's medication history, including the use of glucose-lowering, antihypertensive, and lipid-lowering drugs, as well as insulin, directly reflects their treatment status and adherence, making it an indispensable component for building dynamic risk models (54).

This structured and unstructured data embedded within EHRs can be efficiently extracted and integrated using AI techniques such as natural language processing, providing high-quality input for multimodal fusion and thereby significantly enhancing the predictive performance of the models. In terms of data storage and retrieval infrastructure, while laboratory data is initially generated in specialized Laboratory Information Systems (LIS), it is typically integrated into the central EHR system via interoperability standards to allow for unified access. Consequently, the EHR and LIS function as distinct but interconnected databases, with the EHR serving as the primary interface for clinical decision-making. However, this integration process may lead to data redundancy, such as duplicates arising from both automated interface transmission and manual clinical entry. To address potential inconsistencies between these sources, data preprocessing pipelines for DR management models must employ rigorous cleaning strategies. These typically involve designating the direct LIS feed as the “source of truth” or applying timestamp-based logic to prioritize the most recent and accurate values, ensuring the fidelity of the data fed into the AI models.

#### 2.2.2 Laboratory data

In addition to routine clinical indicators, laboratory data can provide supplementary information for DR risk assessment. These metrics reflect the impact of the systemic metabolic state on retinal pathology and represent potential novel biomarkers (55). Among them, renal function indicators are particularly closely associated with DR. Multiple studies have confirmed that blood urea nitrogen is a significant risk factor for DR (56), with elevated levels, especially above 20 mg/dl, significantly increasing the risk of DR (57). Serum creatinine has also been identified as a key indicator in several predictive models (58). Moreover, urinary microalbumin and the serum uric acid to creatinine ratio have shown significant correlations with DR. Notably, some research has found that in certain patient populations, a higher estimated glomerular filtration rate is also associated with an increased risk of DR (59).

In recent years, research on inflammation-related hematological indicators has offered a new perspective on DR risk prediction, with composite markers such as the systemic immune-inflammation index (SII) and systemic inflammatory response index (SIRI) being particularly prominent. Studies have confirmed that SII is a sensitive indicator for predicting complications and mortality in patients with type 2 DR (60). Furthermore, both SII and SIRI are independent risk factors for DR, and their combined use can significantly improve diagnostic accuracy (AUC = 0.782), showing great potential in the diagnosis and stratified management of DR (61). Crucially, studies have shown that levels of NLR, SII, and SIRI are already significantly elevated before the appearance of clinically visible DR lesions and are negatively correlated with subclinical microvascular damage in the retina detected by SS-OCTA (62). As DR progresses, these inflammatory markers also show a stepwise increase, with SII demonstrating a significant linear relationship with the onset and development of DR, serving as an independent risk factor at all stages of the disease (63).

In summary, laboratory data not only provide important biological explanations for the onset and progression of DR but also serve as a key input for AI models. By quantifying systemic renal function and inflammatory status, they help to achieve early identification and individualized prediction of DR risk.

### 2.3 Emerging data sources

With the advent of the precision medicine era, “omics” data—represented by genomics, proteomics, and metabolomics—are gradually becoming a new dimension for constructing a panoramic view of disease. As previously mentioned, significant individual heterogeneity exists in the onset and progression of DR, with genetic susceptibility considered an important contributing factor, accounting for as much as 25%−50% of the risk of DR onset (64). Genome-wide association studies have identified multiple DR susceptibility loci, but their associations often exhibit ethnic specificity. For example, a Brazilian study found that specific polymorphisms in the TIE2 and ANGPT-1 genes are associated with protection against DR (65), whereas new susceptibility genes identified in a Japanese study, STT3B and PALM2, have not been validated in other populations (64). Similarly, a variation in the PRMT1 gene was found to be associated with an increased incidence of PDR in Japanese patients with type 2 DM (66). Meta-analyses have further consolidated these findings, clarifying that polymorphisms in cytokine genes (such as IFN-γ rs2430561 and TGF-β rs1800471) are associated with a reduced susceptibility to DR (67), and that specific SNPs (such as rs2010963, rs833061) are associated with the onset and progression of NPDR and PDR (68). A recent review pointed out that the genetic polymorphisms of DR are mainly concentrated in key genes such as VEGF, ACE, and APOE (69). Integrating this information into a genetic risk score can serve as an independent data modality for AI models, helping to explain differences in individual prognoses under similar clinical conditions.

In contrast to the static perspective of genomics in revealing genetic susceptibility, proteomics and metabolomics can provide a dynamic snapshot of the disease state at the molecular level, making them of great interest as they are closer to the actual disease phenotype. In the field of metabolomics, researchers are dedicated to finding characteristic metabolites that reflect the onset and progression of DR. Progress has been made in studies on easily accessible biological samples such as serum: a cross-sectional study found that reduced levels of circulating L-Tyrosine can serve as an indicator of the presence of retinopathy in patients with T2DM (70); while a large-scale longitudinal study identified 17 metabolites associated with the risk of incident DR, noting that various N-lactoyl amino acids increase the risk, whereas citrulline is associated with a reduced risk (71). In addition to blood samples, research has also extended to non-invasive specimens like tear fluid, with one study finding that elevated levels of lactate in the tears of PDR patients can serve as an independent risk factor for assessing PDR (72). At the same time, comprehensive studies have revealed dysregulation in multiple pathways in patients with DR, including arginine-proline metabolism, amino acid metabolism, and purine metabolism. These findings provide important clues for elucidating the pathological mechanisms of DR and exploring new therapeutic targets (73).

In the field of proteomics, analysis of intraocular fluids can provide direct insights into the local pathophysiology of the retina. As the vitreous humor is directly adjacent to the retina, changes in its proteome can effectively reflect changes in retinal homeostasis. Studies have shown that proteins related to coagulation, the complement system, and the kallikrein-kinin system are significantly upregulated in the vitreous humor of patients with PDR and can serve as potential biomarkers (74). Proteomic analysis of the aqueous humor has also identified a large number of differentially expressed proteins associated with processes such as inflammation, oxidative stress, and apoptosis, including apolipoprotein A-I, selenoprotein P, and cystathionine β-synthase (75). These findings collectively confirm the complex pathophysiological network of DR, involving local immune inflammation, angiogenesis, coagulation dysfunction, and tissue repair (76).

Although the acquisition costs and analytical complexity of “omics” data are currently high, they represent the future direction of personalized medicine. The multimodal fusion of this deep molecular information with clinical and imaging data has the potential to enable AI models to break through existing bottlenecks and achieve a more precise prediction of DR risk.

## 3 Key technologies and methodologies of multimodal AI

After constructing a multimodal database encompassing ophthalmic imaging, systemic clinical information, unstructured medical texts, and emerging “omics” data, the core challenge lies in effectively integrating these data, which vary in origin, complexity, and dimensionality. The goal is to achieve informational complementarity and synergy, ultimately enhancing the characterization of the disease state and the assessment of clinical risk. Multimodal fusion is a key approach to solving this problem. In recent years, the development of relevant computational methods has provided new ideas and tools for this field, gradually making DR management based on multimodal data a possibility.

### 3.1 Overview of fusion strategies

The fundamental purpose of multimodal fusion is to establish a computational framework that can collaboratively analyze information from different sources to improve the accuracy and reliability of specific clinical tasks. Based on the stage at which information is integrated within the model, classic fusion strategies can be divided into early fusion, late fusion, and hybrid fusion (77). Beyond these traditional architectures, the integration of Large Language Models (LLMs) represents a cutting-edge evolution in multimodal analysis, particularly for processing unstructured text-image pairs, introducing a new paradigm for clinical decision support.

#### 3.1.1 Early fusion

Early fusion, also known as feature-level fusion, is the most direct strategy for multimodal integration. The basic idea is to concatenate or combine features from different modalities at the initial stage of analysis to form a unified high-dimensional feature vector, which is then fed into a downstream predictive model for training (78). For instance, in the CAD-EYE system developed by Khalid et al., the authors employed an early fusion approach by combining feature vectors extracted from two distinct deep learning architectures, MobileNet and EfficientNet. This integration of features at the encoding stage allowed the model to achieve a high classification accuracy of 98% for multi-eye disease diagnosis, surpassing single-model baselines (79). Similarly, Ejaz et al. utilized a parallel CNN framework where deep features extracted from fundus images were fused using Canonical Correlation Analysis before the classification step. This feature-level integration enabled the model to capture more discriminative patterns, resulting in a detection accuracy of 93.39% (80). Hervella et al., through a self-supervised pre-training method, enabled a model to simultaneously learn both common and unique features between multimodal images, thereby constructing a powerful unified feature encoder. This encoder performed exceptionally well in the subsequent DR grading task, validating the great potential of the early fusion strategy in enhancing the model's generalization ability for downstream tasks and addressing the issue of sparse data annotation (81). The advantage of this method is its ability to integrate information from different modalities at the outset of modeling, thereby capturing potential cross-modal correlations and interaction patterns, which helps to improve model performance.

However, its limitations should not be overlooked. First, differences in time scales, spatial resolutions, and data structures across modalities make data alignment difficult, and simple concatenation may introduce noise or cause information loss. Second, discrepancies in feature scales can lead to high-dimensional modalities (such as imaging data) dominating the model, thereby overshadowing important information from other modalities. Finally, this strategy is highly dependent on data completeness; if one modality is missing, the stability and applicability of the overall model will be affected.

#### 3.1.2 Late fusion

Late fusion, also known as decision-level fusion, operates opposite to early fusion. The approach involves first building separate, independent models for each modality, each of which outputs a prediction or decision score based on its own data. Subsequently, at the final stage of analysis, these outputs are integrated using methods such as weighted averaging, majority voting, or a small meta-learner to arrive at a final conclusion (82). A classic application of this strategy is the ensemble approach proposed by Qummar et al., where five independent CNNs—ResNet50, InceptionV3, Xception, Dense121, and Dense169—were trained on fundus images. The final DR detection result was derived by calculating the weighted average of the probability scores from these distinct models, yielding higher robustness than any single model (83). Furthermore, a representative application of this strategy is the DRCNN-Lesion Proxy framework proposed by Sekar et al. In this architecture, global image-level features extracted by a ResNet34 backbone and lesion-specific cues simulated by a proxy module are processed independently and then integrated through a late fusion classification head. By fusing these heterogeneous information sources at the final decision stage, the model achieved robust DR severity prediction with an accuracy of up to 98.37% across multiple public datasets (84). The advantage of this strategy lies in its greater flexibility and modularity. Researchers can select the most appropriate analytical method for each modality based on its characteristics, and the system can still operate on the remaining modalities when some data are incomplete, thus exhibiting good robustness. However, its limitation is that information is integrated only at the decision level, ignoring potential interactions and synergies between modalities during the feature-learning stage. For example, the pathological link between retinal microvascular morphology and neural layer thickness often cannot be effectively utilized in a late fusion framework, thereby limiting the model's ability to uncover deeper patterns of the disease mechanism.

#### 3.1.3 Hybrid/deep fusion

To combine the deep interaction of early fusion with the flexibility of late fusion, hybrid or deep fusion strategies have become a frontier in multimodal research. The core idea of these methods is to abandon a “one-off” integration at the model's input or output, and instead achieve multiple, in-depth information exchanges at the intermediate layers of the model (82). A typical architecture usually involves building separate encoders for each modality or data source to learn their respective feature representations, with connections established between different layers of the networks to enable the dynamic flow and deep integration of cross-modal information. This paradigm aims to introduce cross-modal collaboration at the early stages of feature learning while preserving the independence and specificity of each modality's processing pathway, with the goal of achieving optimal performance.

The superiority of hybrid fusion has been demonstrated in DR research. When processing OCTA data from different retinal depths, a study by Ebrahimi et al. (85) clearly indicated that, compared to early fusion, late fusion, and single-layer inputs, an “intermediate fusion” architecture that fuses features from the superficial, deep, and choriocapillaris layers at the network's middle layers could increase DR classification accuracy to 92.65%, achieving the best performance. Similarly, to integrate the complementary information from different fields of view in OCTA, Li et al. designed a hybrid fusion framework to jointly analyze high-resolution (6 × 6 mm^2^) and ultrawide-field (15 × 15 mm^2^) OCTA images. The results showed that the performance of this fusion strategy in detecting all stages of DR (including early and late) was significantly superior to algorithms using only a single field of view, proving the effectiveness of fusing local fine structures with global vascular layout information (86). Furthermore, the design of hybrid architectures is becoming increasingly sophisticated. Tseng et al. proposed a “two-stage early fusion” model that mimics the diagnostic workflow of an ophthalmologist. The model first performs lesion detection and then severity grading. This sequential hybrid method identified early DR more accurately than traditional algorithms, demonstrating the potential of hybrid fusion in enhancing model robustness and credibility (87).

In practice, achieving the aforementioned deep fusion relies on several advanced computational methods, with the attention mechanism, Transformer, and Graph Neural Network (GNN) being the most representative. The attention mechanism, through cross-modal query, allows a model to dynamically and selectively focus on relevant regions in one modality (e.g., fundus images) based on features from another modality (e.g., high-risk indicators in EHRs), achieving intelligent information weighting (88). The Transformer, with its powerful self-attention mechanism, can effectively capture and fuse long-range dependencies in both time-series and image-series data, thereby accurately characterizing the dynamic evolution of the disease (89). Some studies have attempted to fuse the local feature extraction capabilities of Convolutional Neural Networks (CNNs) with the global modeling capabilities of Vision Transformers (ViTs), achieving high-precision prediction for DR grading (90). Meanwhile, the GNN offers a structured fusion perspective. It models data from different modalities as nodes in a graph and learns their intrinsic connections through a message-passing mechanism. This allows for the systematic characterization of a “panoramic patient profile” that includes imaging, clinical, and “omics” information, thereby generating higher-level disease representations.

#### 3.1.4 Large language models and text-image integration

The integration of LLMs and Vision-Language Models represents a significant leap in processing the unstructured textual component of multimodal data. Unlike traditional NLP methods that rely on rigid rule-based extraction, LLMs demonstrate a superior ability to understand complex clinical narratives and facilitate conversational decision support. However, their direct application in high-stakes medical diagnosis requires critical validation against established deep learning baselines.

Current evidence suggests that while LLMs excel in interaction, they may struggle with precision in specialized diagnostic tasks compared to dedicated models. For instance, a recent study by Rossi et al. compared a WE-LSTM (Word Embedding-Long Short-Term Memory) network against a WizardLM-powered chatbot (DiabeTalk) for diabetes diagnosis. The results revealed a stark contrast: while the LLM-based chatbot offered a user-friendly conversational interface and “acceptable results” without specific training, the specialized WE-LSTM model significantly outperformed it in diagnostic accuracy (97.80 vs. 77.56%) and specificity (95.90 vs. 57.80%) when applied to minimally pre-processed data (91).

This highlights a crucial limitation in current multimodal frameworks: general-purpose foundation models exhibit strong logic and language understanding, but they cannot yet replace specialized, fine-tuned predictive models for precision tasks without extensive domain-specific adaptation. Therefore, the current frontier involves integrating the reasoning capabilities of LLMs with the precision of specialized architectures (like CNNs for images or LSTMs for sequential clinical data) to create systems that are both accurate and communicatively competent.

### 3.2 Explainable AI (XAI)

Although multimodal AI models show great potential in the management of DR, their clinical translation still faces a key challenge: the inherent “black box” nature of deep learning models (92). In a high-risk field like medicine, an AI system that cannot clearly articulate its internal decision-making logic is unlikely to gain the full trust and adoption of clinicians. Therefore, the transparency and interpretability of the decision-making process are necessary prerequisites for promoting the widespread deployment of AI models in clinical settings and realizing their applied value (93). The development and application of XAI technologies, which aim to open the “black box” and clarify the contribution weights of key modalities and features, are a crucial step in transforming multimodal AI from a “high-performance prediction tool” to a “trustworthy clinical decision partner.”

#### 3.2.1 Explanation for visual modalities

When processing core visual modalities like fundus images, explanation methods based on Class Activation Mapping are the most widely used, with Gradient-weighted Class Activation Mapping and its variants becoming mainstream tools. These techniques generate “saliency heatmaps” that intuitively highlight the key image regions upon which a convolutional neural network relies for DR grading or lesion segmentation. Sharma et al. (94) used Grad-CAM to verify that their model was indeed focusing on true pathological areas; Rautaray et al. (95) employed Grad-CAM++ to clearly display the key retinal structures that influenced DR severity grading. Such visual validation methods provide clinicians with an effective review tool, allowing them to quickly determine whether a model's judgment is based on true pathological features like microaneurysms and exudates, thereby enhancing trust in the model's diagnostic logic (96).

More importantly, the value of XAI extends far beyond validation to aiding scientific discovery. A groundbreaking study used Guided Grad-CAM technology to explore sex differences in DR, finding that the model focused more on the macular region when identifying female patients and more on the optic disc and peripheral vessels for male patients. This discovery led to a new clinical hypothesis: female DR patients may be more prone to developing macular edema, while males face a higher risk of proliferative DR (PDR) (97). This fully demonstrates that XAI can not only “explain AI” but also inspire new human understanding of the disease itself.

#### 3.2.2 Explanation for multimodal fusion

For complex models that fuse multimodal data such as fundus images, clinical indicators, and “omics” information, model-agnostic XAI methods offer more powerful explanatory capabilities, capable of revealing the interactions between different modalities. Local Interpretable Model-agnostic Explanations (LIME) approximates the local behavior of a complex model with a simple one by making perturbations around a single sample, thereby revealing the prediction mechanism for that sample (98). In a multimodal context, LIME can clearly quantify the contribution of different data modalities and their internal features to the prediction for an individual patient, providing an intuitive basis for personalized decision-making.

Meanwhile, the game theory-based SHAP (SHapley Additive exPlanations) method has been widely used in recent multimodal DR research due to its ability to provide both local and global explanations and its solid theoretical foundation. For each individualized prediction, SHAP can precisely calculate the marginal contribution of each input feature to the final prediction, clearly revealing the key factors driving individual risk (99). Recent studies have fully demonstrated the great potential of SHAP in DR biomarker discovery: Zong et al. (100) used SHAP to explain their XGBoost model and successfully identified various metabolites (such as C18:1OH, threonine) associated with DR risk and their risk cutoff values. Another study, also applying SHAP, found that glucose, glycine, and age were important predictors across all stages of DR, while creatinine and various phosphatidylcholines showed higher importance in the late PDR stage, suggesting they could be potential biomarkers for severe DR (101). Gui et al. combined machine learning with SHAP to explore the relationship between heavy metal exposure and DR. Their analysis clearly indicated that, among numerous variables, urinary antimony level was the most critical factor influencing predicted DR risk, with a contribution weight far exceeding other variables. This finding not only reveals the potential impact of environmental pollutants on DR but also provides a new direction for early non-invasive screening (102). These cases strongly demonstrate that methods like SHAP can help researchers accurately trace the source from high-dimensional, complex multimodal data to locate key driving factors, with their value extending from mere “model explanation” to “aiding knowledge discovery.”

In general, XAI is a core technology that empowers the clinical translation of multimodal AI in DR management. Its value is reflected on three levels: first, building clinical trust by ensuring that the AI's decision-making process can be reviewed and understood through transparent, intuitive explanations; second, deepening disease understanding by helping researchers gain insights into potential pathophysiological mechanisms from complex data correlations; and third, driving scientific discovery by serving as an efficient biomarker discovery engine to identify new risk factors from high-throughput data.

The organic combination of a high-performance multimodal fusion model and a powerful XAI explanation system will together form the core of the next-generation intelligent management platform for DR. Such a platform will not only provide accurate diagnoses and risk predictions but also clearly reveal why, providing solid and powerful support for achieving truly precise and personalized clinical decisions.

## 4 Frontier explorations and future directions of multimodal AI in DR management

As a complex microvascular complication, the management of DR is a continuous process that includes risk prediction, diagnostic staging, progression monitoring, treatment decision-making, and long-term follow-up (103). By integrating data from different sources, multimodal methods provide new possibilities for establishing a precise and individualized decision support system covering the entire course of DR management (77). As described below, multimodal AI has significant application potential in five key stages of DR management.

### 4.1 Precise risk stratification and early warning

The success of DR management is critically dependent on the early stages (104). Therefore, the primary step in DR management is to accurately identify high-risk individuals before the onset of clinical signs to implement proactive prevention and intervention. Traditional risk models often rely on a few clinical variables and struggle to capture complex individual risks. By integrating data from various sources, multimodal AI can construct more comprehensive and dynamic risk prediction models, achieving a paradigm shift from “disease detection” to “risk prediction.”

A highly representative direction is the fusion of EHR data with fundus images. EHRs contain long-term patient data on blood glucose, blood pressure, laboratory tests, and medication history, which constitute a time-series reflecting their systemic health status. One study combining fundus images with heterogeneous EHR data showed that its fusion model significantly outperformed single-source models in the task of screening for referable DR (AUC of 97.96%), demonstrating that fusing multi-source data can lead to earlier and more accurate referral decisions (105). Another study (106) delved deeper by utilizing 20 years of EHR data to create thousands of features capturing the dynamic evolution of patient health. The multimodal model they built showed outstanding performance in early DR detection (AUC of 0.988), confirming that the systemic health trajectory recorded in EHRs contains rich predictive information even before identifiable lesions appear in the fundus.

Another key strategy is the fusion of multiple ophthalmic imaging modalities to enhance screening efficacy. Liu et al. evaluated the effect of adding self-imaging OCT to traditional FP. The results showed that the combined FP and SI-OCT strategy was significantly superior in both sensitivity (87.69%) and specificity (98.29%) for detecting DME compared to FP alone. More importantly, a cost-effectiveness analysis confirmed that this combined approach is highly advantageous economically, providing reliable support for the early detection and precise referral of DR (107).

To effectively mine the complex relationships between different risk factors, researchers have also developed more advanced multimodal fusion architectures. For example, the VisionTrack framework innovatively introduces a Graph Neural Network to process clinical risk factors, treating different factors as nodes in a graph to learn their potential high-order, non-linear interactions. The framework further integrates a Convolutional Neural Network for processing images and a large language model for parsing clinical notes, significantly improving prediction accuracy by fusing diverse data sources and providing a more comprehensive assessment of retinal health (108).

### 4.2 Comprehensive diagnosis and refined staging

The accurate diagnosis and refined staging of DR are the cornerstones for formulating subsequent treatment and management strategies (109). Multimodal data fusion is a core strategy for enhancing the diagnostic performance of AI, and its advantages have been confirmed in studies integrating various types of imaging, clinical, and even biological sample information. At the imaging level, studies have confirmed that combining multiple imaging modalities can provide complementary pathological perspectives (110). For example, one study that simultaneously analyzed CFP and FFA and extracted key quantitative features using a curvelet transform built an SVM classifier that achieved 100% sensitivity and specificity in a three-level DR staging task (111). At the level of fusing imaging and clinical data, Sandhu et al. evaluated the diagnostic performance for NPDR by fusing clinical data, OCT, and OCTA. The results showed that after integrating clinical data, the model's accuracy reached 96% and its AUC reached 0.96, far surpassing the performance of any single modality (112). Even more innovatively, research has combined proteomics data from non-invasive biological samples (such as tear fluid) with fundus images, demonstrating that this cross-disciplinary data fusion can further improve the sensitivity and specificity of DR diagnosis (113).

To effectively utilize multimodal data, researchers have developed various advanced AI methods. To address the challenge of a scarcity of large-scale annotated data in clinical settings, self-supervised learning has become an important breakthrough. Some research has used multimodal data like fundus images and FFA for self-supervised feature learning, achieving diagnostic performance comparable to supervised models and providing an effective solution for data-scarce scenarios (114). Hervella et al. (81) developed a self-supervised pre-training method that significantly improved the accuracy of the subsequent DR severity grading task by learning both the common and unique features between different modalities.

In terms of model architecture, researchers have designed various sophisticated networks to achieve efficient fusion. For example, a modality-specific attention network designed specific attention modules for CFP and OCT images to learn their complementary information (115). The TFA-Net model, through a twofold feature augmentation mechanism, effectively fused CFP with wide-field SS-OCTA images, showing excellent performance on small datasets (116). In addition, a multimodal information bottleneck network utilizes multicolor imaging technology to simultaneously extract features from multiple modalities to improve DR detection accuracy (117).

As a key component of refined staging, retinal vessel segmentation also benefits from multimodal AI, effectively overcoming the challenges faced by traditional methods (118). For example, CMFNet effectively solved the problem of discontinuous microvessel segmentation by fusing 3D volumetric data from OCTA with 2D projection maps (119). M3B-Net utilized the richer information from FFA images to assist and improve the accuracy of vessel segmentation on UWF images (120). The ELEMENT method innovatively used vessel connectivity as a key feature for machine learning classification, effectively reducing segmentation inconsistencies (121).

Finally, a systematic review and meta-analysis covering 47 studies ultimately confirmed that deep learning-based multimodal methods demonstrate high accuracy in DR detection and have the potential to be reliable automated diagnostic tools in the clinic (122). In summary, through data fusion, methodological innovation, and the application of key technologies, multimodal AI is driving the diagnosis and staging of DR toward greater precision and comprehensiveness.

### 4.3 Dynamic disease progression prediction

DR is a chronic, progressive disease. Accurately predicting the probability and time window for a specific patient to progress from non-proliferative DR (NPDR) to PDR or to develop DME is crucial for seizing the optimal moment for intervention and preventing irreversible vision loss (123). However, static, single-time-point examinations are insufficient to capture the dynamic evolution of the disease. Multimodal AI, especially models capable of processing time-series data, offers a possible path toward achieving dynamic disease progression prediction (124).

Currently, researchers have explored and validated the potential of AI in predicting DR progression from multiple dimensions. At the clinical imaging level, studies have confirmed that deep learning models can not only predict DR progression over 2 years with high accuracy using only baseline fundus images (125), but can also accurately predict disease evolution over the next 5 years by analyzing a series of retinal images in multi-ethnic datasets (126).

At the molecular biology level, research has begun to delve into the predictive information within gene expression data. For example, one study analyzed single-cell transcriptomics data from the fibrovascular membranes of PDR patients, identified differential gene expression patterns in specific cells, and built a high-accuracy machine learning prediction model based on these findings (AUC of 0.83–0.96), providing a new avenue for predicting PDR risk from a molecular dimension (127).

Furthermore, some cutting-edge research is dedicated to visualizing the future evolution of the disease using AI. For example, the DRForecastGAN framework uses generative adversarial network technology to synthesize potential future fundus images based on a patient's current images. The model shows superior accuracy in predicting future DR severity (AUC of 0.85) and can intuitively “depict” the progression of the disease, providing an innovative visual tool for patient-doctor communication and treatment decisions (128).

To integrate data from the aforementioned different sources and dimensions to build more powerful predictive models, researchers are exploring more advanced fusion architectures. The Transformer is considered an ideal choice for this task due to its outstanding ability to process sequential data and capture long-range dependencies, making it suitable for fusing longitudinal EHR data, series of images, and even molecular biomarkers. A Transformer-based multimodal model can not only analyze cross-modal associations at each point in time but also learn the disease's evolution pattern along the entire timeline, thereby providing clinicians with a dynamic, forward-looking view for disease management.

### 4.4 Personalized treatment decision support

With the popularization of treatments such as anti-VEGF drugs, a new era has begun for the treatment of DR. However, there is significant heterogeneity in how different patients respond to the same treatment regimen. Therefore, accurately predicting a patient's response to a specific treatment is key to achieving personalized medicine (129). By deeply mining pre-treatment data, multimodal AI has the potential to identify biomarkers that are predictive of treatment response.

Imaging biomarkers are an important basis for predicting treatment response. One study using quantitative UWFA found that baseline vascular leakage patterns could predict the speed and duration of the response to anti-VEGF therapy: lower diffuse leakage predicted a faster response, while a higher ratio of perivascular to diffuse leakage predicted a more lasting effect (130). In addition, the RetmarkerDR model, by calculating the dynamic imaging metric of “microaneurysm turnover,” can not only assist in judging DR progression but also be used to evaluate treatment efficacy, providing a new tool for quantifying therapeutic effects (131).

Molecular biomarkers provide deeper biological information for prediction. A pioneering study integrated metabolomics and lipidomics data from aqueous humor samples and, combined with machine learning, successfully identified metabolites that could effectively predict a strong therapeutic response. The model they built can accurately screen for potential weak responders (132). Similarly, by analyzing the angiogenic properties of specific proteins (such as decorin) in the aqueous humor, the efficacy of anti-VEGF drugs can also be predicted (133).

Looking to the future, an ideal personalized treatment decision model for DR will integrate baseline imaging, molecular biomarkers, and patient EHR and genomics data. By training on large data cohorts and combining with XAI techniques, the model will not only be able to predict efficacy but also explain the reasons, thereby providing clinicians with direct and actionable decision support.

### 4.5 Intelligent follow-up interval recommendation

Optimizing follow-up management is a core issue in the long-term care of DR, directly related to the effective use of medical resources and the patient's visual prognosis (109). Current follow-up intervals are mostly based on static disease staging guidelines and do not fully consider the individualized risk of the patient. This can lead to the over-treatment of low-risk, stable patients and insufficient follow-up for high-risk, progressive patients (134). By conducting a comprehensive and dynamic risk assessment of the patient, multimodal AI can provide data-driven support for recommending personalized follow-up intervals, achieving an optimal allocation of medical resources.

An intelligent follow-up recommendation system is essentially a top-level application built on the aforementioned risk stratification and progression prediction models. The DeepDR Plus system is a prime example; its research showed that through AI-based personalized risk assessment, the average screening interval could be extended from the standard 12–31.97 months, while reducing the delayed detection rate of DR progression to 0.18% (135). This strongly demonstrates the feasibility and superiority of dynamically adjusting screening intervals. Based on such evidence, some studies have even suggested that for T2DM patients without DR, the screening interval could be safely extended to 2–3 years (134).

The decision logic of such intelligent systems is dynamic and multidimensional: first, the system will conduct an initial risk assessment based on a risk stratification model and recommend a longer initial follow-up interval for low-risk individuals. Second, at each follow-up visit, the system will use a dynamic progression prediction model, inputting the latest multimodal data to update the patient's probability of future worsening events. If the probability significantly increases, the system will automatically issue an alert and recommend shortening the follow-up interval. Finally, for patients undergoing treatment, the aforementioned treatment response model can evaluate the efficacy of the treatment and adjust the follow-up plan accordingly.

In this way, multimodal AI transforms DR follow-up management from a population-based, static model to an individual-based, dynamic, and adaptive one. This model not only ensures that high-risk patients receive the most timely monitoring and intervention, preventing vision loss due to delays, but also significantly improves the efficiency and cost-effectiveness of the healthcare system by reducing the unnecessary clinical burden on low-risk, stable patients, ultimately achieving a win-win for both patient benefit and medical resource optimization (136).

### 4.6 Critical assessment: clinical feasibility vs. experimental frontiers

While the potential of multimodal AI is vast, a critical distinction must be drawn between technologies that are currently feasible for clinical deployment and those that remain in the experimental “proof-of-concept” phase. Currently, unimodal AI systems based on fundus photography have reached a high level of maturity, with several achieving regulatory approval and real-world implementation. These systems benefit from standardized imaging protocols, clear ground truths, and established reimbursement pathways.

In contrast, the “panoramic” multimodal frameworks discussed in this review—particularly those integrating multi-omics, longitudinal EHRs, and foundation models (LLMs)—are primarily experimental. As evidenced by the comparison between WE-LSTM and WizardLM, the integration of LLMs for clinical decision support faces significant hurdles regarding “hallucinations,” lack of interpretability, and a performance gap compared to specialized networks in structured tasks. Furthermore, the infrastructure required to unify disparate data sources in real-time does not yet exist in most healthcare settings (91). Therefore, while multimodal fusion represents the theoretical future of precision medicine, its immediate clinical utility is currently limited to research hospitals with integrated data lakes, whereas widespread adoption awaits the resolution of interoperability standards and rigorous prospective validation of these complex, heterogeneous models.

## 5 Challenges faced

Although multimodal AI shows great potential in the “panoramic” management of DR, successfully translating it from theoretical research into a widely used clinical tool still requires overcoming multiple challenges at the data, technical, and application levels (103, 137). Clearly understanding these challenges and planning future directions accordingly is crucial for promoting the healthy and sustainable development of this field.

### 5.1 Data-level challenges

High-quality, large-scale, and standardized multimodal data are the core driving force behind the development of AI models. However, there are currently three major bottlenecks at the data level (138). First is the issue of data silos and sharing difficulties. Multimodal data from DR patients are often stored dispersedly in different hospitals, departments, and even different information systems, forming hard-to-surmount “data silos.” Strict patient privacy regulations and institutional data barriers make large-scale, multi-center data integration extremely difficult. The lack of effective data sharing mechanisms greatly limits the scale and diversity of data needed for model training, which is one of the most common limitations in current research (139). Second is the problem of standardizing multi-center data. Different medical institutions use different imaging equipment, acquisition protocols, image quality standards, and formats and terminologies for recording clinical data (140). This heterogeneity leads to a severe data distribution shift, causing models trained at one center or in a specific clinical trial to often fail to achieve the same excellent performance on data from another center or in the real world, which severely affects the model's generalization ability (141). Finally, there is a lack of high-quality public datasets. Currently, most datasets used for multimodal ophthalmic AI research are limited in scale and are often private (142). As numerous literature reviews have pointed out, there is a general lack of large-scale public datasets freely available to researchers in the field of ophthalmic AI (143). There is a particular scarcity of large-scale, public, multimodal datasets that include long-term follow-up information in longitudinal cohorts. While cross-sectional data can be used to develop diagnostic models, longitudinal data are indispensable for the crucial task of “progression prediction” in DR management (144).

### 5.2 Technical-level challenges

At the algorithm and model level, there are also difficult hurdles to overcome. The first is the generalization ability and robustness of the models. As mentioned earlier, due to data heterogeneity, many AI models are at risk of “overfitting,” performing excellently on the training dataset but experiencing a sharp decline in performance when encountering low-quality images, rare lesions, or different ethnic populations in a real clinical environment (145). Developing robust models that can maintain stable and reliable performance in complex and variable clinical scenarios is a key focus of current technical research. The second is the limitation of causal inference. Current deep learning models are essentially powerful “pattern recognizers,” excelling at discovering complex correlations in data, but not causality. Prediction models based on correlation pose potential risks when guiding clinical interventions and therefore must be interpreted with caution and validated with prospective studies. Finally, there is the need for the deepening of XAI models. Although techniques like Grad-CAM provide preliminary tools for visual explanation, they are mostly focused on explaining a single modality. For multimodal models, we not only need to know which region of an image the model is focusing on but also understand how it balances and integrates information from different modalities. Developing XAI methods that can clearly reveal the cross-modal decision-making logic is key to building clinical trust and promoting the true integration of models into the decision-making process (146).

### 5.3 Application-level challenges

Successfully deploying multimodal AI into clinical practice still faces numerous real-world obstacles. First is the challenge of seamless integration with clinical workflows. Busy clinical workflows have extremely high demands for efficiency. An AI system that requires doctors to manually upload multiple types of data, is complex to operate, and has long waiting times is unlikely to be accepted (147). Designing a user-friendly system that can automatically pull data from hospital information systems, run analyses in the background, and present results in a simple and intuitive way is key to determining whether the technology can be implemented. Second is the need for strict regulatory approval. As medical devices, AI software must undergo rigorous approval processes by agencies such as the National Medical Products Administration or the U.S. Food and Drug Administration (148). Compared to unimodal diagnostic support software, the validation and approval process for multimodal AI systems used for prediction and management is more complex and takes longer. Furthermore, there is the issue of cost-effectiveness analysis. The purchase of multimodal imaging equipment and the deployment of AI systems require significant upfront investment (147). Healthcare institutions and health policy decision-makers need to see clear evidence that this investment will bring long-term economic benefits through early intervention, reduced costs for treating complications, and optimized allocation of medical resources (149). Finally, there are challenges related to physician acceptance and ethical issues. Gaining the trust of clinicians is at the core of AI application (150). This depends not only on XAI technology but also requires large-scale prospective clinical trials to prove its effectiveness and safety in the real world. In addition, ethical and legal issues, such as potential algorithmic bias and the attribution of responsibility for medical errors, also need to be fully discussed and resolved before the technology is widely promoted (151, 152).

## 6 Conclusion and outlook

Multimodal AI is beginning a profound transformation, with the potential to reshape the management of DR by promoting a fundamental paradigm shift from standardized grading based on static, single-source information to personalized decision-making that integrates multi-source, dynamic data. By fusing ophthalmic imaging, systemic data, and emerging “omics” information, multimodal AI can construct an unprecedented “panoramic” digital model for each patient. This has the potential not only to significantly improve the accuracy of early warning, precise staging, and dynamic progression prediction for DR but also to provide a solid data foundation for formulating personalized treatment strategies.

Although the application of multimodal AI in the DR field is still in an exploratory stage, facing multiple challenges such as the scarcity of high-quality longitudinal datasets, the need to validate model generalization, and unclear clinical translation pathways, this has not diminished its great potential as a core driving force for future precision ophthalmology. Future research should focus on constructing large-scale, standardized multimodal public databases, developing more efficient and explainable fusion algorithms, and conducting prospective clinical validation studies to accelerate the translation of technology from the lab to the clinic.

In summary, multimodal AI is not merely an improvement on existing diagnostic tools but a transformative force that can run through the entire process of DR management. It provides a novel perspective and powerful tools for conquering the challenge of blindness caused by this complex disease.
